# The Effect of Virtual Mindfulness-Based Interventions on Sleep Quality: A Systematic Review of Randomized Controlled Trials

**DOI:** 10.1007/s11920-021-01272-6

**Published:** 2021-07-23

**Authors:** Amanda Jiang, Michael Rosario, Sara Stahl, Jessica M. Gill, Heather L. Rusch

**Affiliations:** 1 National Institute of Nursing Research, National Institutes of Health, Bethesda, MD USA; 2 Washington University School of Medicine, St. Louis, MO USA; 3 TEACCH Autism Program, University of North Carolina at Chapel Hill, Chapel Hill, NC USA; 4 National Institutes of Health, 3 Center Drive, Building 3, Room 5E/26, Bethesda, MD 20892 USA

**Keywords:** Virtual, Mindfulness, Meditation, Sleep quality, Insomnia

## Abstract

**Purpose of Review:**

We summarized peer-reviewed literature investigating the effect of virtual mindfulness-based interventions (MBIs) on sleep quality. We aimed to examine the following three questions: (1) do virtual MBIs improve sleep quality when compared with control groups; (2) does the effect persist long-term; and (3) is the virtual delivery method equally feasible compared to the in-person delivery method?

**Recent Findings:**

Findings suggest that virtual MBIs are equivalent to evidence-based treatments, and to a limited extent, more effective than non-specific active controls at reducing some aspects of sleep disturbance. Overall, virtual MBIs are more effective at improving sleep quality than usual care controls and waitlist controls. Studies provide preliminary evidence that virtual MBIs have a long-term effect on sleep quality. Moreover, while virtual MBI attrition rates are comparable to in-person MBI attrition rates, intervention adherence may be compromised in the virtual delivery method.

**Summary:**

This review highlights virtual MBIs as a potentially effective alternative to managing sleep disturbance during pandemic-related quarantine and stay-at-home periods. This is especially relevant due to barriers of accessing in-person interventions during the pandemic. Future studies are needed to explore factors that influence adherence and access to virtual MBIs, with a particular focus on diverse populations.

## Introduction

A recent meta-analysis (*n* = 54,231) reported that disrupted sleep affects approximately 40% of the general and healthcare populations during the novel coronavirus pneumonia (COVID-19) pandemic [1••]. Several factors contribute to the rising rate of disrupted sleep, including decreased physical activity from home quarantine, increased screen time from teleworking, and the barrage of stress-inducing news related to the pandemic [2•,^3^•]. Pandemic-induced poor sleep quality is concerning, given that sleep disturbances are associated with increased risk for adverse physical health outcomes [4, 5], mood dysregulation [6, 7], decreased cognitive function [8, 9], and compromised immune system [10, 11]. Moreover, sleep quality is directly linked to vaccine immune response [12]. As such, addressing sleep disturbance during the pandemic is critical for population well-being.

Pharmaceutical sleep aids and behavioral therapy (e.g., cognitive behavioral therapy (CBT)) are two forms of evidence-based treatments available to address poor sleep. However, medication can cause adverse side effects (e.g., memory loss, behavioral changes, headaches) and result in abuse and dependence [13, 14]. While CBT is more tolerable, it can be expensive, inaccessible, and may not be a viable standalone treatment for all patients [15–17]. For instance, individuals with substantial sleep deprivation (i.e., total sleep time < 3.65 h) may not be able to endure certain components of CBT, such as sleep restriction, and usually require additional time commitment to experience improvement in sleep quality [16, 18]. In this case, adjunctive strategies are needed to promote retention and encourage a clinical response. Furthermore, the structure of CBT may not be feasible for populations that have a greater propensity for decreased motivation (e.g., individuals facing multiple mental and physical health conditions) [15]. As such, alternative approaches are needed to increase patient choice and sustain treatment engagement. Mindfulness-based interventions (MBIs), such as mindfulness-based stress reduction (MBSR) and acceptance and commitment therapy (ACT), have demonstrated efficacy in improving sleep quality and may serve as an alternative treatment for sleep disturbance [19, 20]. Mindfulness training involves a non-judgmental awareness of the present moment [21]. MBIs consist of 1- to 2-month instructional courses that include weekly mindfulness exercises and lectures. While there are many MBIs with different lecture material and supplementary content, they incorporate a common set of exercises involving formal meditation (e.g., sitting meditations, walking meditations, body scans) and informal practices (e.g., cultivating awareness in daily activities such as eating, breathing, and walking). Traditionally, MBIs take place in-person and in a community-based setting [22]. Due to the pandemic, traveling to and from wellness destinations and participating in large group gatherings are limited in safety and feasibility.

Technological advances have enabled the virtual delivery of behavioral interventions across a range of personal devices, which can circumvent some of the challenges encountered with in-person delivery of behavioral interventions during a pandemic [23]. For instance, virtual MBIs, which are delivered via an online or offline program and accessed via technological devices, such as laptops or mobile phones, have the potential to increase accessibility. Examples of virtual MBI program interfaces include self-guided web-based applications and therapist-led video conference sessions. Research comparing the efficacy of virtual MBIs to in-person MBIs suggests that the two delivery methods are comparable. For example, Wolever and colleagues [24] found that virtual and in-person MBIs produced similar improvements in stress, as well as sleep quality. They also observed lower rates of attrition in the virtual mindfulness group (3.8%) compared to the in-person mindfulness group (27.3%), suggesting that the virtual delivery method may be more feasible. On the other hand, a study comparing teleconference-based ACT to in-person ACT found that both delivery methods improved physical and mental health symptoms, but not sleep disturbance [25]. Given the increased disruption of sleep and social distancing guidelines resulting from the COVID-19 pandemic, there is a significant need to test the efficacy of sleep interventions that are accessible from the home. The objective of this study is to systematically review randomized controlled trials that employed a virtual MBI in populations with clinically significant sleep disturbance. Furthermore, to assess for relative efficacy, comparator groups (i.e., specific active controls (evidence-based treatments), nonspecific active controls, usual care controls, and waitlist controls) were analyzed separately. We aim to examine the following three questions: (1) do virtual MBIs improve sleep quality when compared with control groups; (2) does the effect persist long-term; and (3) is the virtual delivery method equally feasible compared to the in-person delivery method?

## Method

### Search Strategies

The review was conducted in accordance with the Preferred Reporting Items for Systematic Reviews and Meta-Analysis (PRISMA) guidelines [26]. PubMed, Scopus, Embase, Cochrane Library, and Web of Science databases were searched for peer-reviewed articles in English through December 3, 2020, with no start date restriction. For search terms, three main subject-heading domains were combined with the AND operator: (online OR internet OR digital OR m-health OR e-health OR computer* OR web* OR app OR smartphone OR mobile application) AND (mindful* OR meditate* OR Vipassana OR “acceptance and commitment therapy”) AND (sleep [TIAB] OR insomnia [TIAB]). The TIAB function was used only for searches in PubMed. Upon completion of the search, two investigators independently examined the title and abstract of each trial to assess for eligibility. Afterwards, the full text article was downloaded for all potentially eligible trials and screened for inclusion. The bibliography of identified trials and germane review articles were manually surveyed for additional references.

### Inclusion and Exclusion Criteria

We included published articles of randomized controlled trials in adults (age ≥ 18 years) with clinically significant sleep disturbance that employed a virtual MBI. Included studies also had assessments of pre-intervention and post-intervention sleep quality. Sleep quality measures included both objective and subjective validated measurements, such as actigraphy, diary-reported sleep quality, and self-reported sleep quality questionnaires: the Insomnia Severity Index (ISI), the Medical Outcomes Study-Sleep Scale (MOS-SS), the Pittsburgh Sleep Quality Index (PSQI), and the Basic Nordic Sleep Questionnaire (BNSQ). The pre-intervention sleep quality mean score (i.e., baseline weighted average) was used to determine if each study cohort had clinically relevant sleep disturbance based on established cut-off scores. Studies with baseline weighted average scores above the minimum established clinical cutoffs for sleep disturbance were included in the review (i.e., ISI > 8; MOS-SS > 20; PSQI > 5) [27–29]. Studies were excluded if they included children, adolescents, and experienced meditators, as well as experimental comparators (e.g., in-person MBIs). Table 1 provides a detailed summary of the inclusion and exclusion criteria.

**Table 1:** Detailed inclusion and exclusion summary

	Inclusion	Exclusion
Population	Adult populations with clinically significant sleep disturbance (i.e., had an ICD insomnia diagnosis or met symptom severity threshold defined by sleep quality questionnaires)	Children, adolescents, and experienced meditators (i.e., ≥ 1000 practice hours)
Intervention	Virtual mindfulness-based interventions (i.e., mindfulness exercises delivered via an online or offline program and accessed via computer, laptop, or other technological devices, such as a tablet or mobile phone) No limit on minimum or maximum duration	In-person mindfulness-based interventions, movement-based therapies (e.g., tai chi and yoga), and integrative therapies (i.e., interventions with multiple modalities and only a small component of mindfulness exercises)
Comparator	Specific active controls (i.e., evidence-based treatments) non-specific active controls, usual care controls, or waitlist controls	Experimental interventions or experimental administration (e.g., comparing app-based mindfulness meditation to in-person mindfulness meditation)
Outcome	Validated subjective or objective measure of sleep quality with pre-intervention and post-intervention assessments	No validated measure of sleep quality or only a pre-intervention assessment
Study design	Randomized controlled trials	Non-randomized controlled trials
Other	Quantitative, peer-reviewed studies reported in English including dates through December 3, 2020, with no start date restriction	Abstracts, reviews, and non-published trials, as well as published trials on duplicate participant samples and in languages other than English

### Data Extraction and Analysis

Two investigators independently extracted data from the eligible articles for further analysis. Data extracted included the author, publication year, population type, sample size, mindfulness intervention features, control intervention features, intervention duration, intervention hours, sleep quality scale, and assessment data. Discrepancies in eligibility and data extraction were resolved through contact with corresponding authors and discussion and consensus with a third investigator. When possible and not reported, standard deviations were calculated using available data. The feasibility of the virtual delivery method was determined by comparing intervention adherence and attrition rates to benchmarks used in clinical trials (i.e., adherence rates > 80% and attrition rates < 20%), as well as adherence and attrition rates from our prior meta-analysis [19], which employed similar inclusion/exclusion criteria using an in-person delivery method. Per protocol definitions of adherence were used and attrition rates were defined as the percent of randomized participants that did not complete the post-intervention assessment.

## Results

### Search Results

**Fig. 1:**
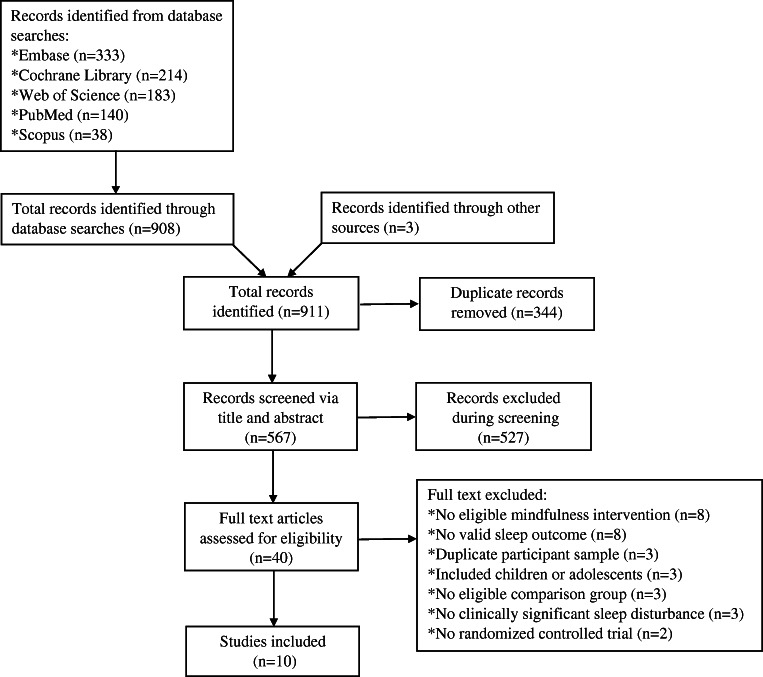
Flow diagram from record identification to final study inclusion

A total of 911 records were initially identified for inclusion in the review. After removing duplicates (*n* = 344), another 527 records were further excluded based on title and abstract. A full-text assessment of the remaining 40 articles was conducted and 10 studies with 2777 participants were included in the review (see Fig. 1, CONSORT flow diagram).

### Characteristics of Included Studies

The major characteristics of the 10 included studies can be found in Table 2. The studies were published from 2012 to 2020, and sample sizes ranged from 21 to 2081. Mindfulness meditation was the most commonly employed MBI (*n* = 4), followed by acceptance and commitment therapy (*n* = 3). The majority of studies used a web-based app as the intervention delivery method *(n* = 8). Expected mindfulness durations ranged from 4 to 16 h over 4 to 8 weeks. All studies included at least one subjective sleep measure, and one study used a combination of subjective and objective sleep measures.

**Table 2:** Study characteristics grouped by control type

Authors, year	Control type	Population	Mindfulness *n*^a^	Control *n*^b^	Mindfulness intervention	Mindfulness intervention delivery mode	Comparison intervention	Comparison intervention delivery mode	Intervention duration	Intervention hours^c^	Scale /V0 mean^d^
Hesser et al., 2012	SAC	Tinnitus	35	32	ACT	Web-based App	CBT	Web-based App	8 weeks	16	ISI 13.91
Low et al., 2020	SAC	Insomnia	12	11	MM	Mobile App	Progressive muscle relaxation	Mobile App	40 or 60 days^e^	6.67 or 10	ISI 13.61
Mak et al., 2017	SAC	Young adults	1041	1040	MM	Web-based App	CBT	Web-based App	8 weeks	5	MOS-SS 26.49
Boettcher et al., 2014	NSAC	Anxiety disorders	45	46	MM	Web-based App	Discussion forum	Web-based App	8 weeks	16	ISI 11.74
Hesser et al., 2012	NSAC	Tinnitus	35	32	ACT	Web-based App	Discussion forum	Web-based App	8 weeks	16	ISI 13.49
Messer et al., 2019	UCC	Cancer survivors	11^f^	10^f^	MBSR	Web-based App	Usual care	NA	6 weeks	8	PSQI 11.57
Simister et al., 2018	UCC	Fibromyalgia	33	34	ACT	Web-based App	Usual care	NA	8 weeks	NR	PSQI 12.97
Chapoutot et al., 2020	Waitlist	Insomnia with hypnotic dependence	16	16	ACT-enhanced CBT	Video-conference	Waitlist	NA	8 weeks	4	PSQI 14.80
Lappalainen et al., 2019	Waitlist	Insomnia	43	43	ACT	Web-based App	Waitlist	NA	6 weeks	NR	ISI 16.96
Nissen et al., 2020	Waitlist	Cancer survivors	104	46	MBCT	Web-based App	Waitlist	NA	8 weeks	NR	ISI 11.73
Querstret et al., 2017	Waitlist	Working adults	63	64	MM	Web-based App	Waitlist	NA	4 weeks	NR	PSQI 11.26

*ACT* acceptance and commitment therapy, *CBT* cognitive behavioral therapy, *ISI* Insomnia Severity Index, *MBCT* mindfulness-based cognitive therapy, *MBSR* mindfulness-based stress reduction, *MM* mindfulness meditation, *MOS-SS* Medical Outcomes Study-Sleep Scale, *NA* not applicable, *NR* not reported, *NSAC* nonspecific active control, *PSQI* Pittsburgh Sleep Quality Index, *SAC* specific active control, *UCC* usual care control

^a^Mindfulness sample size is reported as participants initially randomized to the intervention group

^b^Control sample size is reported as participants initially randomized to the control group

^c^Mindfulness hours are reported as expected hours per intervention, excluding home practice

^d^The V0 sleep quality mean score (i.e., baseline weighted average) was used to determine if each study cohort had clinically relevant sleep disturbance based on established cut-off scores

^e^Due to time constraints, some participants were asked to complete 40 days of mindfulness meditation instead of 60 days; both groups were combined in the analysis.-

^f^Two participants dropped out of the study after randomization. The study did not report the two participants’ group affiliation; thus, the sample sizes reflect the number of participants who completed the intervention

### Change in Sleep Quality from Pre- to Post-intervention

Herein, we briefly report the results of the 10 randomized controlled trials that met our eligibility criteria, stratified by control group type. Table 3 provides a more comprehensive depiction of the results.

**Table 3:** The relative effect of virtual mindfulness-based interventions on sleep quality outcomes

Authors, year	Sleep quality outcome	Mindfulness pre-intervention mean (SD)	Mindfulness post-intervention mean (SD)	Within-group effect size/*P*-value	Control pre-intervention mean (SD)	Control post-intervention mean (SD)	Within-group effect size/*P*-value	Between-group effect size^b^/*P*-value	Group × Time effect size^b^/*P*-value
**Specific active controls**									
Hesser et al., 2012	ISI	13.23 (5.80)	8.48 (5.43)	NR ***p*** **< 0.05**	14.66 (6.30)	9.93 (6.85)	NR ***p*** **< 0.05**	NR NR	NR *p* > 0.16
Low et al., 2020	ISI	13.33 (3.47)	7.67 (3.28)	*d* = 1.61 ***p*** **< 0.05**	13.91 (1.97)	7.73 (4.03)	*d* = 1.97 ***p*** **< 0.05**	NR NR	*η*_*p*_^*2*^ = 0.003 *p* = 0.79
	ATG-TIB	476.31 (161.94)	520.80 (87.70)	*d* = 0.34 *NS*	510.80 (37.56)	511.00 (48.75)	*d* = 0.00 *NS*	NR NR	*η*_*p*_^*2*^ = 0.33 *p* = 0.28
	ATG-TST	436.60 (42.28)	447.30 (87.11)	*d* = 0.15 *NS*	411.00 (88.81)	428.40 (39.59)	*d* = 0.25 *NS*	NR NR	*η*_*p*_^*2*^ = 0.46 *p* = 0.86
	ATG-SOL	10.30 (5.80)	3.00 (2.07)	*d* = 1.67 ***p*** **< 0.05**	5.70 (5.20)	5.60 (3.83)	*d* = 0.02 ***p*** **< 0.05**	NR NR	*η*_*p*_^*2*^ = 0.02 ***p*** **= 0.02**
	ATG-WASO	70.50 (20.89)	64.40 (23.48)	*d* = 0.27 ***p*** **< 0.05**	91.00 (81.43)	75.90 (24.64)	*d* = 0.26 ***p*** **< 0.05**	NR NR	*η*_*p*_^*2*^ = 0.01 *p* = 0.86
	ATG-SE	83.00 (5.00)	86.20 (3.60)	*d* = 0.73 *NS*	83.40 (8.88)	83.80 (3.40)	*d* = 0.05 *NS*	NR NR	*η*_*p*_^*2*^ = 0.37 *p* = 0.51
	ATG-SFI	24.90 (4.70)	25.20 (5.50)	*d* = 0.05 *NS*	25.30 (8.00)	23.30 (7.04)	*d* = 0.25 *NS*	NR NR	*η*_*p*_^*2*^ = 0.58 *p* = 0.46
	SD-TWT	68.10 (44.77)	51.01 (21.24)	*d* = 0.48 ***p*** **< 0.05**	46.42 (30.09)	25.22 (19.34)	*d* = 0.83 ***p*** **< 0.05**	NR NR	*η*_*p*_^*2*^ = 0.005 *p* = 0.77
Mak et al., 2017	MOS-SS	26.03 (19.91)	17.58 (37.60)	*d* = 0.44 ***p*** **< 0.05**	26.92 (19.65)	19.13 (38.53)	*d* = 0.41 ***p*** **< 0.05**	NR *p* > 0.05	NR *p* = 0.91
**Nonspecific active controls**									
Boettcher et al., 2014	ISI	12.20 (5.70)	7.30 (4.70)	*d* = 0.82 ***p*** **< 0.05**	11.30 (6.10)	9.20 (6.30)	*d* = 0.45 ***p*** **< 0.05**	*d* = 0.36 *p* = 0.75	NR ***p*** **= 0.02**
Hesser et al., 2012	ISI	13.23 (5.80)	8.48 (5.43)	NR ***p*** **< 0.05**	13.78 (6.54)	11.22 (6.97)	NR NR	*d* = 0.42 NR	NR *p* > 0.10
**Usual care controls**									
Messer et al., 2019	PSQI	10.57 (3.45)	8.50 (3.14)	NR NR	12.67 (3.07)	12.75 (3.02)	NR NR	*d* = 1.14 ***p*** **< 0.01**	NR NR
Simister et al., 2018	PSQI	12.67 (3.80)	10.24 (3.60)	NR ***p*** **< 0.05**	13.26 (3.80)	13.00 (3.47)	NR ***p*** **< 0.05**	*d* = 0.79 ***p*** **= 0.02**	NR *p* = 0.06
**Waitlist controls**									
Chapoutot et al., 2020	ISI	21.00 (4.40)	10.70 (5.20)	*d* = 1.90 ***p*** **< 0.05**	19.20 (3.90)	17.20 (3.50)	NR NR	NR NR	*d* = 1.98 ***p*** **< 0.001**
	PSQI	14.70 (4.10)	9.10 (3.90)	NR NR	14.90 (3.10)	12.30 (2.80)	NR NR	NR NR	*d* = 0.82 ***p*** **= 0.02**
	SD-TST	6.60 (0.30)	6.50 (0.20)	NR NR	6.00 (0.20)	6.30 (0.20)	NR NR	NR NR	*d* = 1.56 *p* = 0.09
	SD-SOL	0.60 (0.10)	0.20 (0.10)	NR NR	0.70 (0.10)	0.60 (0.10)	NR NR	NR NR	*d* = 1.48 *p* = 0.29
	SD-NWAK	1.40 (0.30)	0.90 (0.40)	*d* = 0.85 ***p*** **< 0.05**	1.30 (0.30)	1.70 (0.40)	NR NR	NR NR	*d* = 0.49 ***p*** **< 0.001**
	SD-WASO	0.80 (0.48)	0.30 (0.30)	*d* = 0.90 ***p*** **< 0.05**	0.60 (0.50)	0.60 (0.50)	NR NR	NR NR	*d* = 3.90 ***p*** **= 0.01**
	SD-SE	76.10 (2.70)	85.20 (2.30)	*d* = 0.69 NR	76.90 (2.60)	79.10 (2.20)	NR NR	NR NR	*d* = 2.58 ***p*** **= 0.03**
Lappalainen et al., 2019	BNSQ^a^	21.77 (4.45)	19.91 (5.25)	NR ***p*** **< 0.05**	21.65 (4.07)	21.57 (4.45)	NR NR	*d* = 0.42 NR	NR ***p*** **= 0.001**
Nissen et al., 2020	ISI	11.70 (5.50)	10.30 (6.00)	*d* = 0.23 *p* = 0.13	11.80 (6.30)	10.10 (6.90)	*d* = 0.26 *p* = 0.25	NR NR	*d* = 0.06 *p* = 0.76
Querstret et al., 2017	PSQI	11.72 (3.81)	8.22 (5.79)	*η*_*p*_^*2*^ = 0.19 ***p*** **< 0.001**	10.79 (5.07)	10.45 (5.07)	*η*_*p*_^*2*^ = 0.38 ***p*** **< 0.001**	*η*_*p*_^*2*^ = 0.16 ***p*** **< 0.001**	NR NR

*ATG* actigraphy, *BNSQ* Basic Nordic Sleep Questionnaire, *ISI* Insomnia Severity Index, *MOS-SS* Medical Outcomes Study-Sleep Scale, *NR* not reported, *NS* not significant, *NWAK* number of awakenings, *PSQI* Pittsburgh Sleep Quality Index, *SD* sleep diary, *SE* sleep efficiency, *SFI* Sleep Fragmentation Index, *SOL* sleep onset latency, *TIB* time in bed, *TST* total sleep time, *TWT* total wake time, *WASO* wake after sleep onset

^a^The study used ISI to screen for sleep disturbance and BNSQ as an outcome measure

^b^Positive effect sizes should be interpreted in favor of the mindfulness group

Effect size (*d*) interpretation: 0.2 = small effect, 0.5 = medium effect, 0.8 = large effect

Partial eta squared (*η*_*p*_^*2*^) interpretation: 0.01 = small effect, 0.06 = medium effect, 0.14 = large effect

*P*-values in bold are statistically significant at < 0.05

#### Specific Active Controls

Three of the included studies used specific active control groups (i.e., evidence-based treatments), with a total of 2171 participants. Hesser et al. [30] and Mak et al. [31] reported significant within-group effects on self-reported sleep quality for the virtual mindfulness and virtual CBT groups, with no significant difference between interventions over time. However, at 1-year follow-up, Hesser et al. [30] found a decreased maintenance effect for the virtual mindfulness group compared to the virtual CBT group. On the other hand, at 3-month follow-up, Mak et al. [31] reported a similar maintenance effect on sleep quality improvement in the virtual mindfulness and virtual CBT groups. Low et al. [32] reported significant within-group effects on self-reported sleep quality, actigraphy-measured sleep onset latency, actigraphy-measured wake after sleep onset, and diary-reported total wake time for the virtual mindfulness and virtual progressive muscle relaxation groups. The virtual mindfulness group also had a greater reduction in actigraphy-measured sleep onset latency over time, relative to the virtual progressive muscle relaxation group. No significant group by time interaction effects were found for the self-reported sleep measures.

#### Non-specific Active Controls

Two of the included studies used non-specific active control groups, with a total of 158 participants. Boettcher et al. [33] reported significant within-group effects on self-reported sleep quality in both the virtual mindfulness and virtual discussion forum control groups; however, the virtual mindfulness group had greater improvements over time, relative to the virtual discussion forum control group. At 6-month follow-up, sleep improvement was maintained in the virtual mindfulness group. On the other hand, Hesser et al. [30] reported a significant within-group effect on self-reported sleep quality in the virtual mindfulness group only, with no significant difference between the virtual mindfulness and virtual discussion forum interventions over time.

#### Usual Care Controls

Two of the included studies used usual care control groups, with a total of 88 participants. Messer et al. [34] reported a large between-group effect on self-reported sleep quality in favor of the virtual mindfulness group. Simister et al. [35] reported significant within-group effects on self-reported sleep quality in both the virtual mindfulness and usual care control groups. Although there was a medium between-group effect in favor of the virtual mindfulness group, the group by time interaction effect was not significant. At 3-month follow-up, the between-group effect on sleep quality was still in favor of the virtual mindfulness group but slightly attenuated (*d* = .53).

#### Waitlist Controls

Four of the included studies used waitlist control groups, with a total of 395 participants. Chapoutot et al. [36] reported small to large group by time interaction effects on self-reported sleep quality and diary-reported number of awakenings, wake after sleep onset, and sleep efficiency, in favor of the virtual mindfulness group. At 6-month follow-up, improvement in self-reported sleep quality was maintained in the virtual mindfulness group; however, improvement in diary-reported sleep measures (number of awakenings and wake after sleep onset) was not maintained. Lappalainen et al. [37] reported a group by time interaction effect on self-reported sleep quality, in favor of the virtual mindfulness group. At 6-month follow-up, improvement in sleep quality was maintained in the virtual mindfulness group. Nissen et al. [38] reported no significant within-group effect on self-reported sleep quality in the virtual mindfulness or waitlist control groups, as well as no significant group by time interaction effect. At 6-month follow-up, there was no change in sleep quality in either group. Querstret et al. [39] reported significant within-group effects on self-reported sleep quality for the virtual mindfulness and waitlist control groups, with a large between-group effect in favor of the virtual mindfulness group. At 3- and 6-month follow-up, sleep quality improvement was maintained in the virtual mindfulness group.

### Feasibility of Virtual Delivery Method

Adherence rates were reported in all but two studies. The definition of intervention adherence varied between studies. The most common definition used was the average number of mindfulness exercises or sessions completed out of the total offered, followed by self-reported engagement with course materials. Due to variations in adherence definitions, we could not compare adherence rates across studies, although rates ranged from 31 to 100%. Two (20%) virtual MBI studies had adherence rates above 80%; meanwhile, seven (39%) in-person MBI studies had adherence rates above this same level [19]. Attrition rates were reported in all but one study, which ranged from 0 to 90%. Six (60%) virtual MBI studies had attrition rates below 20%; similarly, 12 (67%) in-person MBI studies had attrition rates below this same level [19].

## Discussion

To our knowledge, this is the first study to systematically analyze the effects of virtual MBIs on sleep quality among populations with clinically significant sleep disturbance. Our findings suggest that virtual MBIs are comparable to evidence-based treatments (e.g., CBT), and to a limited extent, more effective than non-specific active controls at reducing sleep disturbance. Overall, virtual MBIs are more effective at improving sleep quality than usual care controls and waitlist controls. Our findings are aligned with other systematic reviews and meta-analyses, which found improvements in stress and psychiatric symptoms in clinical and general populations following virtual MBIs [40•,^41^•,^42^•]. Our findings, in conjunction with prior literature, have global implications for improving mental and physical health during a pandemic and could inform the implementation of virtual MBIs during physical distancing, quarantine, and lockdown periods.

Of the eight studies that included follow-up assessments, six studies reported a maintenance effect at follow-up, providing promising evidence that virtual MBIs have a long-term effect on sleep quality improvement. However, two findings warrant further investigation. First, it is unclear whether virtual MBIs are comparable to evidence-based treatments at sustaining sleep improvement long term. While Mak et al. [31] reported a similar maintenance effect for virtual mindfulness meditation and virtual CBT, Hesser et al. [30] observed a decreased maintenance effect for virtual acceptance and commitment therapy compared to virtual CBT. The discrepancy could be attributed to intervention characteristics and sample characteristics, including the severity of sleep disturbance. Second, a maintenance effect may be dependent on the type of sleep measurement used. At follow-up, Chapoutot et al. [36] found a maintenance effect on self-reported sleep quality, but not on diary-reported sleep measures. As such, virtual MBIs may provide more lasting benefits on some aspects of sleep but not all. For long-term and well-rounded benefits, participants may need to practice mindfulness exercises for longer durations and beyond study requirements. Sustained involvement is critical in mindfulness training, as continuity of practice is deemed essential for cultivating and enhancing well-being [22, 43].

Adherence rates ranged widely in the reviewed studies (31 to 100%), which were lower than our prior meta-analysis that examined the effects of in-person MBIs in sleep-disturbed populations [19]. Possible explanations for the lower adherence rates observed in virtual MBIs may be related to lack of therapist guidance, distractions in the home environment, or technical problems accessing the intervention [42•, 44, 45]. Caution is needed when interpreting these findings due to heterogeneity in adherence definitions used across studies, which ranged from computer-measured minutes spent on course materials to self-reported hours spent completing course materials. Only one study included in the review examined the relationship between adherence and changes in sleep quality score [34], finding a positive correlation (*r* = 0.39; *p* = 0.042). However, it is important to recognize that improvements in sleep quality may not be due entirely to greater adherence rates. Prior research suggests that for some participants a shorter period of intense engagement in virtual MBIs may be enough to prompt behavior change, while other individuals may require prolonged and personalized training [46, 47•]. As such, this may explain the association between low adherence rates and improved sleep quality demonstrated in some of the studies reviewed. Attrition rates also ranged widely in the reviewed studies, with one large-scale study conducted by Mak et al. [31] reporting an attrition rate of 90%. With the exception of Mak et al. [31], the attrition rates for the other reviewed studies (0 to 31%) are lower than the rates reported by a prior study that examined virtual MBIs in clinical and nonclinical populations (7.7 to 52.3%) [48] and are similar to our prior meta-analysis that examined the effects of in-person MBIs in sleep-disturbed populations (0 to 50%) [19]. There are characteristics that seem to correlate with lower attrition rates; two reviewed studies identified younger age, male gender, increased well-being at baseline, higher treatment expectancy, and prior experience with mind-body interventions as key factors [31, 38].

The current findings shed light on important clinical implications that could inform the implementation and dissemination of virtual MBIs during a pandemic. Findings indicate that virtual MBIs may be as effective as evidence-based treatments and may alleviate some aspects of moderate to severe sleep disturbances. From a clinical standpoint, virtual MBIs could complement other established sleep treatments or serve as second-line treatments when first-line treatments are not effective or available [49••]. The accessibility and flexibility of virtual MBIs allow patients and the general public to have increased agency for their own care [50•]. For individuals who are infected with SARS-CoV-2 and have limited access to mental health professionals, they can safely access MBIs via virtual platforms in quarantine or isolation rooms. On a population level, continuous access to effective self-guided care may help prevent further psychological and physiological harm imposed by a global health crisis.

The findings should be interpreted in the context of several limitations. First, the review summarized studies that were not focused on evaluating virtual MBIs during a pandemic. As such, the reader needs to interpret the findings with caution, given that the pandemic has imposed additional stressors [51], which may influence treatment efficacy. This caution was previously raised by Fischer et al. [49••], who also used pre-pandemic data to extrapolate the efficacy of self-guided interventions on sustaining mental well-being during the pandemic. Second, we examined randomized controlled trials with various types of control groups, including usual care controls and waitlist controls, raising the probability that the reported benefits could be the result of nonspecific effects (e.g., expectations). Despite limitations associated with using these types of control groups, usual care controls and waitlist controls remain ethical approaches to evaluating the effects of mind-body therapies [52]. Third, we were only able to identify a few studies in each control group type, which prevented us from conducting a meta-analysis that would have otherwise provided more precise estimates of study efficacy. Fourth, measurements of adherence varied greatly and lacked standardization in the reviewed studies, making comparison between studies challenging. Finally, since females constituted the majority of participants (except in one study), the generalizability of the results to other demographics, such as men and those identified as nonbinary, is limited. This discrepancy is consistent with a meta-analysis of mindfulness studies in nonclinical populations, which found that 56 to 100% participants were women (mean = 78%) [53]. Even though virtual MBIs have the potential to reach wider populations, utilization will be limited if only certain populations are willing to engage in or have access to virtual MBIs.

Future studies should investigate the impact of adherence rates on symptom improvement. The development of a standardized adherence definition may aid in comparison across studies. Additional investigation is needed to understand how to reduce attrition rates in targeted populations (e.g., healthcare providers), increase engagement in men, and remove barriers for marginalized groups that may not have access to technology. The review only identified one study that used a combination of subjective and objective sleep measures. Future research could employ more objective measures to investigate the effect of virtual MBIs on objective sleep factors, such as actigraphy-measured sleep onset latency and total sleep time, which can offer insights into specific sleep parameters that virtual MBIs target. Some studies included additional intervention strategies, such as cognitive and behavioral strategies, leading us to question whether mindfulness-based components have added benefits. This should be addressed in future studies that thoroughly dismantle multi-component interventions.

## Conclusion

Overall, this review provides evidence that suggest virtual MBIs can be beneficial in improving some aspects of sleep quality in adults with sleep disturbance. Given that the prevalence of sleep disturbance is increasing during the COVID-19 pandemic and that in-person interventions have become largely infeasible, virtual MBIs may be an effective alternative to manage sleep abnormalities, or a potential addition to standard telehealth care. Further investigation is needed to explore factors that influence adherence and access to virtual MBIs, with a particular focus on diverse populations.

## Data Availability

Not applicable.
